# New Herbal Biomedicines for the Topical Treatment of Dermatological Disorders

**DOI:** 10.3390/biomedicines8020027

**Published:** 2020-02-08

**Authors:** Julia Hoffmann, Fabian Gendrisch, Christoph Mathis Schempp, Ute Wölfle

**Affiliations:** Research Center Skinitial, Department of Dermatology, Medical Center, University of Freiburg, Hauptstr. 7, D-79104 Freiburg, Germany; julia.hoffmann@uniklinik-freiburg.de (J.H.); fabian.gendrisch@uniklinik-freiburg.de (F.G.); christoph.schempp@uniklinik-freiburg.de (C.M.S.)

**Keywords:** atopic dermatitis, acne, actinic keratoses, psoriasis, rosacea, wound healing

## Abstract

Herbal extracts and isolated plant compounds play an increasing role in the treatment of skin disorders and wounds. Several new herbal drugs, medicinal products and cosmetic products for the treatment of various skin conditions have been developed in recent years. In this nonsystematic review, we focus on herbal drugs that were tested in controlled clinical studies or in scientifically sound preclinical studies. The herbal biomedicines are intended to treat atopic dermatitis (St. John’s wort, licorice, tormentil, bitter substances, evening primrose), psoriasis (araroba tree, lace flower, barberry bark, indigo, turmeric, olibanum, St. John’s wort), actinic keratosis (birch bark, petty spurge), herpes simplex (lemon balm, sage and rhubarb), rosacea (green tea, licorice, tormentil) and acne vulgaris (tea tree oil, green tea, hop), or to improve photo protection (green tea, Dyer’s weed, cocoa tree, carotinoids, licorice), aesthetic dermatology (licorice, pine bark, gotu kola) and wound healing (birch bark, onion).

## 1. Introduction

Herbal therapies have been used for the treatment of skin conditions for centuries. Several plant compounds are still used in topical treatments, such as salicylic acid from willow bark from *Salix* spp. (for desquamation), 8-methoxypsoralen from *Ammi visnaga* (L.) Lam. (for photochemotherapy), and tannins from oak bark, black tea or hamamelis bark (for oozing eczema). Traditionally used medical plants were evaluated and documented in 300 monographs by Commission E at the German institute for drugs and medicinal products (BfArm) between 1976 and 1993. About 30% of these plants received a negative evaluation. The positive monographs contained 25 plants with relevance for dermatological treatments. They include well-known medical plants such as chamomile, which hazel and marigold. However, most of these plants only achieved a low level of evidence for their efficacy, because only a few high quality clinical studies have been performed [1,2]. During the last years the therapeutic potential of medical plants traditionally used in dermatology has been explored, and some of them have been developed and approved as drug or medical device for the treatment of skin disorders. Furthermore, an increasing number of herbal products have been developed in the field of medical cosmetics, often called “cosmeceuticals”. This review highlights the most exciting new experimental and clinical studies with herbal drugs and cosmeceuticals for skin disorders. Literature was searched in actual plant reviews focusing on special skin diseases. We focused predominantly on controlled clinical studies with a sufficiently high level of evidence. However, this is not purely a systematic evidence based review, because we have also included scientifically confirmed effects of plant extracts with promising new treatment options for dermatologists. In this way we could point to new auspicious treatment strategies, although clinical studies are still missing or under preparation. The quality of the clinical studies was classified as “levels of evidence” (LOE) A to D according to the proposal of the UK National Health Service [3]. Level A represents the highest level with randomized, controlled clinical studies and cohort studies. Level B includes conclusive retrospective or analytical cohort studies, research results, and case control studies, as well as follow-up studies of level A. Level C includes case reports or follow-up studies of level B and level D is assigned to expert opinions without scientific background, pure laboratory research or mechanisms of action. The outline of the paper is arranged according to dermatological conditions and at the end of this article, the main findings are summarized.

## 2. Atopic Dermatitis

Atopic dermatitis (AD) is a chronic, pruritic inflammatory skin disease. Dermatologists often prescribe glucocorticoids to the patients, but patients and parents of children with AD worry about the side effects of glucocorticoids, especially in long term therapy. They ask for herbal therapies because they expect similar effectivity and fewer side effects. A comprehensive, evidence-based review on clinical studies with herbal products for AD has been published recently [4]. Some of the studies are highlighted here.

### 2.1. St. John’s Wort (Hypericum perforatum (L.))

St. John’s wort is traditionally used as hypericum oil for the treatment of wounds and burns. The lipophilic phloroglucin derivative hyperforin displays antibacterial, anti-inflammatory and keratinocyte differentiation-promoting properties [5]. The efficacy of a hyperforin-rich ointment standardized to 1.5% hyperforin has been investigated in a randomized, placebo-controlled half side comparison pilot study in 21 AD patients. The treatment was performed twice daily over a period of 4 weeks [6] (LOE-A).

### 2.2. Licorice (Glycyrrhiza glabra (L.))

The anti-inflammatory effect of licorice (*Glycyrrhiza glabra* L. and *Glycyrrhiza uralensis* Fisch. ex DC.) is well studied and summarized in an actual review [7]. Most studies were performed with the triterpenes glycyrrhizin and glycyrrhetinic acid of licorice on skin [7,8,9,10]. However, other ingredients, such as the flavonoid isoliquiritigenin [11] and the chalcone licochalcone A [12,13,14] display also anti-inflammatory effects. A hydrophilic ointment with 2% glycyrrhetinic acid as the main active ingredient was tested in a randomized, placebo-controlled study on 281 adult subjects with mild to moderate AD. The application was performed three times daily in the affected areas. After five weeks the verum was significantly superior to the vehicle (placebo) (80% vs 10% improvement) [15] (LOE-A). In a placebo controlled study with 26 adults a cream with Licochalcone A as anti-inflammatory ingredient showed anti-inflammatory effects superior to placebo [16] (LOE-A). Furthermore it has been shown that a herbal composition with glycyrrhizinic acid (0.6%) and licorice extract (0.1% *Glycyrrhiza uralensis* root extract) as main active ingredients displays anti-inflammatory effects in a placebo-controlled double-blind UV-erythema test study 48 h after irradiation and cream application. The licorice-based product was as effective as 1% hydrocortisone acetate [17]. Besides, it reduced the severity score in 10 patients with AD treated twice daily over 2 weeks in a non-interventional pilot study [17] (LOE-B).

### 2.3. Tormentil (Potentilla erecta (L.))

Tannins from black tea (*Camellia sinensis* (L.) Kuntze), witch hazel (*Hamamelis virginiana* L.) and oak bark (*Quercus* spp.) have been empirically used in dermatology since ancient times. Tannins are used as wet-lipid wraps or local baths for the treatment of acute, oozing eczema. A cream containing 2% tannins from the rhizome of tormentil (*Potentilla erecta* (L.) Raeusch.) displayed a corticoid-like vasoconstrictive effect in an occlusive patch test after 48 h [18]. It also displayed placebo-controlled anti-inflammatory properties comparable to hydrocortisone in the UV-erythema test (LOE-A) and was effective in the treatment of 24 patients suffering from mild to moderate AD. The application was performed twice daily over 2 weeks, and was not placebo controlled [19] (LOE-B).

### 2.4. Bitter Substances

Bitter substances have been used as appetizing and digestion promoting agents since Ayurvedic medicine 5000 years ago. Only recently the molecular structure of bitter taste receptors (TAS2Rs) has been elucidated, and it was shown that TAS2Rs are also expressed in human epidermis [20]. Bitter compounds such as salicin from willow bark (from *Salix* spp.) and amarogentin from *Gentiana lutea* (L.) bind to the bitter taste receptors of the skin, eventually leading to calcium influx and the enhanced expression of skin barrier-constituting proteins such as filaggrin [20]. Bitter compounds also stimulated the synthesis of lipids in keratinocytes. In a placebo-controlled, double-blind half-side comparison with 33 volunteers 5% gentian extract significantly increased the lipid content of the epidermal stratum corneum on the volar forearm. In this body region skin lipids are nearly exclusively produced by keratinocytes [21]. The application occurred twice a day for 4 weeks. After 2 weeks of treatment, a significant increase in the lipid content could already be observed (LOE-B). Interestingly, the predilection sites of AD (flexures of the arms and knees) correspond to skin regions in which the lipids are exclusively formed by keratinocytes and not by sebaceous glands. Therefore, topical treatment with bitter agents is especially helpful in dry and atopic skin with reduced synthesis of epidermal lipids.

### 2.5. Evening Primrose (*Oenothera* biennis (L.))

The oil obtained from evening primrose seeds is beneficial for AD due to its high content of γ-linolenic acid. It is used both internally and in topical products. Only a few high-quality studies have investigated the effect of evening primrose oil in AD. A recent meta-analysis of the existing literature concludes that there is a moderate effect of evening primrose oil on itching, scaling and crusting in AD [22] (LOE-A).

## 3. Psoriasis Vulgaris

Herbal products are also used for the topical treatment of psoriasis. Psoriasis is a chronic, immune-mediated skin disease that shows red and scaly patches on the skin that itch or burn. Three systematic reviews have evaluated the use of herbal therapies in psoriasis [23,24,25].

### 3.1. Araroba Tree (Vataireopsis araroba (Aguiar) Ducke)

The most potent topical treatment for psoriasis is the anthracen derivative dithranol (synonym: anthralin). It was obtained from chrysarobin, extracted from the bark of the araroba tree that grows in the rain forests of the Amazon. Dithranol inhibits the release of pro-inflammatory cytokines and the proliferation of keratinocytes. A randomized controlled multicenter study on 106 psoriasis patients with chronic psoriasis plaques demonstrated significant better therapeutic effectivity for dithranol short contact treatments (15–45 min) with stepwise increasing concentrations of dithranol (up to 5%) once daily for 12 weeks as compared to the standard treatment calcipotriol ointment (50 µg/g twice daily) [26] (LOE-A).

### 3.2. Lace Flower (Ammi majus(L.) and Ammi visnaga (L.))

The furanocoumarins 8-methoxypsoralen (8-MOP) and 5-methoxypsoralen (5-MOP) are isolated for therapeutic use from *Ammi majus* (L.) and *Ammi visnaga* (L.) Lam. The psoralens are phototoxic substances that are photo-activated by long-wave ultraviolet A (UVA) radiation and may cause severe phototoxic skin reactions. In the therapeutic setting of PUVA therapy (psoralen plus UVA) they inhibit keratinocyte proliferation and display immunosuppressive effects that are used to treat severe inflammatory skin conditions, i.e., psoriasis. Several clinical studies confirmed the efficacy of systemic PUVA [27] (LOE-A), bath PUVA [28] (LOE-A) and cream PUVA therapy (0.1% 8-MOP) [29] in psoriasis (LOE-A).

### 3.3. Barberry Bark (Mahonia aquifolium (Pursh) Nutt.)

The barberry *Mahonia aquifolium* is a shrub indigenous to Northern America. It was used for centuries by Native Americans to treat psoriasis. Tinctures and ointments from *Mahonia* bark are available as traditional drugs in Northern America and Europe. Recently, a randomized placebo-controlled double-blind study in 200 psoriasis patients demonstrated the efficacy and safety of a 10% *Mahonia* ointment in the treatment of psoriasis. The application was done twice daily for 12 weeks [30] (LOE-A).

### 3.4. Indigo (Baphicacanthus cusia, Brem.)

‘Indigo naturalis‘ is an important remedy in Traditional Chinese Medicine (TCM). It is a blue powder obtained from the plant *Baphicacanthus cusia* by grinding, fermentation and addition of lime. In a randomized placebo-controlled study 42 patients suffering from chronic plaque psoriasis were treated once daily with a 10% indigo containing ointment for 12 weeks. The indigo naturalis used contained 1.4% indigo and 0.16% indirubin. Treatment with indigo improved symptoms by 81%, while the improvement with placebo was only 26% [31] (LOE-A). As a side effect, four patients showed itching. Because Indigo naturalis causes long lasting blue staining of skin and clothes, Taiwanese researchers developed a patented uncolored Indigo naturalis extract [32]. Several studies with Indigo extract have been performed in psoriasis. A recently published randomized, double-blind, placebo-controlled study in 100 psoriasis patients showed a dose-dependent efficacy of Indigo extract applied twice a day over 8 weeks. Indigo extract (200 µg/g) reduced the PASI by 70%, and Indigo extract (50 µg/g) reduced the PASI by 50%. Side effects in some patients were nasopharyngitis, infections of the upper respiratory tract and local erythema. Severe side effects were not observed [33] (LOE-A). Punch biopsies obtained before treatment and after 8 weeks of treatment revealed a normalization of skin morphology and downregulation of the pro-inflammatory key cytokine in psoriasis, IL-17 [33]. All studies with Indigo extract were performed on Asian patients. Whether the effect of Indigo naturalis is comparable in Caucasians cannot be assessed.

### 3.5. Turmeric (Curcuma longa (L.))

Turmeric plays an important role in TCM and in Aryuvedic Medicine. In vitro, turmeric and its major active ingredient curcumin display anti-inflammatory, antimicrobial and anti-oxidative properties [34]. During the last years some laboratory and clinical studies have investigated the therapeutic potential of curcumin in psoriasis. Curcumin may improve psoriasis by inhibition of phosphorylase kinase [35,36], downregulation of pro-inflammatory cytokines such as IL-17 and TNF-α, as well as improvement of the epidermal barrier by inducing the expression of involucrin and filaggrin in vitro [37]. However, randomized placebo-controlled studies with turmeric and curcumin in psoriasis are missing so far [38].

### 3.6. Olibanum (Boswellia Serrata, Triana & Planch.)

Olibanum containing ointments were recommended in the Greco-Roman period by Hippocrates, Galen and Dioscorides for the treatment of various skin disorders such as psoriasis, burns, warts, bleeding and wounds. Recently, 200 patients with mild to moderate psoriasis were treated three times daily for 12 weeks with an olibanum ointment containing 5% 3-O-Acetyl-11-keto-β-boswellic acid in an open label application study. The PASI was significantly reduced, as well as serum biomarkers such as leukotrien B4, TNF-α, VEGF and PGE_2_ (LOE-B). Thirteen patients (6.5%) developed contact dermatitis [39].

### 3.7. St. John’s Wort (Hypericum perforatum (L.))

Psoriatic keratinocytes show increased cell proliferation, disturbed cell differentiation, an inflammatory phenotype and reduced expression of cationic channels such as TRPC6. Hyperforin—the major lipophilic active ingredient of St. John’s wort—displays in vitro pronounced anti-inflammatory effects and stimulates calcium influx into psoriasis keratinocytes, activates TRPC6 expression, reduces cell proliferation and promotes proper cell differentiation [40]. A placebo-controlled single-blind pilot study with 5% St. John’s wort extract on 10 psoriasis patients revealed a significant improvement of the erythema, extension and thickness of the psoriasis plaques. The treatment was performed twice daily [41] (LOE-B). Similarly, a recently published placebo-controlled, double-blind half-side comparison in 20 patients with mild to moderate plaque psoriasis with 5% St. John’s wort extract showed a significant reduction of the PASI and the lesional TNF-α expression [42] (LOE-B).

## 4. Herpes Simplex

The herpes simplex virus (HSV) can cause blisters and sores in almost any part of the skin. These sores usually occur either around the mouth and nose, or on the genitals and buttocks. Several extracts were described with antiviral activity [43] including licorice extract [44] and *Boswellia serrata* oleo gum [45]. However clinical studies using these plant products are still missing. Here, we focused on plants with clinical evidence against herpes simplex infection.

### 4.1. Lemon Balm (Melissa officinalis (L.))

Lemon balm cream was tested in 66 patients with recurrent *herpes simplex labialis* in a randomized double-blind, placebo-controlled study. The application of the test cream (1% dried *Melissa officialis* extract) was four times daily for 5 days. The lesions cleared significantly faster with lemon balm cream, and the patients had less blisters and pain [46] (LOE-A).

### 4.2. Sage (Salvia officinalis (L.)) and rhubarb (Rheum palmatum (L.))

A randomized, placebo-controlled study investigated the combination of sage and rhubarb extract in 149 patients with *herpes simplex labialis*. The patients were treated topically with either acyclovir (50 mg/g), sage extract (23 mg/g), or the combination of sage and rhubarb extract (23 mg/g each). The combined sage-rhubarb cream was superior to sage alone, and was as effective as acyclovir [47] (LOE-A).

## 5. Actinic Keratosis

Actinic keratoses (AK) represent in situ squamous cell carcinomas that form on sun-damaged skin and appear clinically as thick, scaly or crusty skin. Several plant extracts were tested against actinic keratosis; however, the results were mostly not promising (e.g., for St John’s wort [48].

### 5.1. Birch Bark (Betula spp.)

Birch bark contains 87% triterpenes, mainly betulin. A betulin oleogel is produced by mixing a standardized dry extract from birch bark with vegetable oil. In a multicenter, placebo-controlled, randomized study with betulin oleogel, 165 patients were treated topically for 3 months and showed no positive effect. However, two thirds of the patients had actinic keratosis grade 3 [49] (LOE-A). In contrast in a non-randomized, non-placebo controlled study with 28 patients with actinic keratosis grade 1 or 2 it could be demonstrated that a therapy with betulin cream (betulin oleogel with additional water) was as effective as cryotherapy [50] (LOE-B). The same result was achieved with betulin oleogel in a prospective, randomized, monocentric study with 45 patients with early grades of AK [51] (LOE-B). Histological and experimental studies suggest a differentiation-promoting effect as main mechanism of action of betulin in early grades of actinic keratosis [51,52].

### 5.2. Petty Spurge (Euphorbia peplus (L.))

The latex of the lactiferous petty spurge contains toxic diterpene esters such as ingenol mebutate. A randomized double-blind, placebo-controlled study evaluated the efficacy of 0.025% and 0.05% ingenol mebutate gel compared to placebo in 200 patients with actinic keratoses. The topical products were applied once daily for three days. Both ingenol mebutate concentrations were highly effective (75% and 100% clearing, versus 0% clearing with placebo). Ingenol mebutate induces a localized necrosis of the treated skin, followed by an inflammatory response, crusting and subsequent clearing of the treated area. Scar formation was not observed [53] (LOE-A). These results were confirmed in another randomized double-blind, placebo-controlled study [54] (LOE-A). Ingenol mebutate gel was approved in 2013 as a prescription drug (150 µg/g gel and 500 µg/g gel).

## 6. Photoprotection and Esthetic Dermatology

Numerous plants contain photo-protective antioxidants and ultraviolet (UV) absorbing polyphenols (catechins and flavonoids), and carotinoids. These compounds may protect the skin from sunburn, skin ageing and the development of skin cancer, that are at least in part mediated by reactive oxygen species (ROS). A systematic review from 2014 summarizes topically and orally provided plant compounds for improved protection from sunlight [55]. Plant extracts are also used for multiple cosmetic indications. A review published in 2010 highlights controlled clinical studies for different cosmetic indications as well as for photo protection [2].

### 6.1. Green Tea (Non-Fermented Camellia sinensis (L.))

Green tea extracts contain high amounts of oligomeric proanthocyanidins such as catechin, epicatechin and epigallocatechin-3-gallate, that are potent antioxidants with photo-protective properties. They reduce oxidative stress induced by UV radiation and inhibit various cytokines and mediators involved in skin carcinogenesis [56] (LOE-D). Some studies have shown that green tea extract may prevent UV-induced inflammation when applied topically or systemically [57]. However, in vivo studies confirming that green tea may prevent skin cancer are still missing.

### 6.2. Dyer’s Weed (Reseda luteola (L.))

Flavonoids such as quercetin, rutin and luteolin are potent antioxidants. Dyer’s weed (is particularly rich in luteolin. It has been shown that Dyer’s weed extracts and luteolin display prominent anti-oxidant and anti-inflammatory effects in skin cells [58]. Additionally, luteolin absorbs UVA radiation and protects skin cells from UVB-induced DNA -damage [59,60]. In a randomized, placebo-controlled, double-blind study with 40 healthy volunteers a Dyer’s weed extract containing 2.5% luteolin reduced UVB-induced erythema to a similar extent as 1% hydrocortisone [61] (LOE-A). It has been recently shown that oxidative stress also plays a critical role in the induction of irritant and allergic contact dermatitis [62]. An ointment containing a combination of luteolin, tocopherol and ubichinone with a high radical protecting factor may protect the skin from skin irritation induced by alkali, acid, alcohol, soap, detergents and disinfection. This protective effect has been proven in the repetitive washing test in a placebo-controlled, double-blind study with 25 volunteers. As active ingredients, the antioxidant cream contained 0.1% Dyer’s weed extract, 0.1% tocopherol and 0.05% ubiquinone. 15 min prior to the washings, 200 μL of the test ointment was applied. The test was performed 3 times daily on 7 consecutive days. The antioxidant ointment significantly reduced erythema and transepidermal water loss and improved skin hydration in the washing test [63] (LOE-A).

### 6.3. Cocoa Tree (Theobroma cacao (L.))

Cocoa powder contains a mixture of flavanols and tannins, mainly catechin and epicatechin. In a randomized comparative double-blind study the photo-protective effect of cocoa consumption on UV-induced skin erythema was assessed in 24 female volunteers. The volunteers ingested cocoa with a high (326 mg/d) or a strongly reduced flavanol content (27 mg/d) dissolved in 100 mL water over 12 weeks. Subsequently, a UV-erythema test was performed in all subjects. It was shown that only the flavanol-rich cocoa preparation reduced the UV-erythema. Moreover, the consumption of the flavanol-rich cocoa drink significantly increased skin firmness and hydration [64] (LOE-A).

### 6.4. Carotinoids

The effect of the plant-derived carotinoids β-carotene and lycopene on UV-induced skin erythema was assessed in a randomized placebo-controlled, double-blind parallel group study with 36 volunteers. On a daily basis, the subjects orally received either 24 mg β-carotene, or a carotinoid mix with 8 mg of β-carotene, lutein and lycopene respectively, or placebo. Skin erythema was measured at the beginning, after 6 weeks and after 8 weeks. Both the carotinoid mix and β-carotene significantly reduced UV-erythema after 8 weeks compared to the placebo [65] (LOE-A). Similarly, a controlled study with lycopene showed a significant reduction of the UV-erythema after 12 weeks [66] (LOE-B). A diet supplementation with a tomato paste containing high concentration in lycopene (16 mg lycopene) was tested in 20 women for 12 weeks in a randomized, controlled, single-blinded study. The assessing investigator was unaware of supplement allocation. A reduced UV-induced MMP-1 (Matrix-Metallo-Proteinase-1) production and protection from mitochondrial DNA-damage was shown in skin samples [67] (LOE-B).

### 6.5. Citrus Fruits (Citrus spp.)

A case-control study with 470 participants showed that the consumption of citrus peel was associated with a lower risk to develop squamous cell carcinoma (SCC) (LOE-C) [68].

### 6.6. Coffee Plants (Coffea spec.)

A prospective observatory study from the “Nurses’ Health Study and Health professionals” with 173229 patients showed that those patients with the highest quintile of caffeine intake compared with those in the lowest quintile had reduces Basal Cell Carcinoma (BCC) risk [69] (LOE-B). A case-control study compared 166 patients with BCC to 158 patients not showing BCC. No significant effect of coffee drinking could be shown on BCC risk [70] (LOE-B).

### 6.7. Licorice (Glycyrrhiza glabra (L.))

The root of licorice contains many different biologically active compounds [7]. The steroid saponin β-glycyrrhetinic acid is the best studied anti-inflammatory compound of licorice. In a randomized, placebo-controlled, double-blind intra-individual comparison study, the effect of an ointment with 2.5% β-glycyrrhetinic acid on the subcutaneous fat tissue of the thigh was investigated in 18 female volunteers. When applied twice daily over 4 weeks, the ointment with β-glycyrrhetinic acid significantly reduced the circumference of the thigh compared to the non-treated or placebo-treated contralateral thigh. The authors hypothesized that topically applied β-glycyrrhetinic acid is absorbed by the skin and may reduce subcutaneous depots of fat tissue through interaction with the local 11-hydroxysteroid dehydrogenase, eventually inhibiting adipocyte growth and maturation [71] (LOE-A).

Licorice extracts are also traditionally used for depigmentation purposes. It has been shown that the lipophilic compound glabridin inhibits the enzyme tyrosin kinase and melanogenesis in melanocytes in animal experiments. The application of an ointment with 0.5% glabridin reduced UVB-induced pigmentation on guinea pig skin [72] (LOE-D).

Some studies have investigated the photo-protective properties of the flavonoid licochalcone A present in licorice roots. In HaCaT keratinocytes licochalcone A upregulated various anti-inflammatory and cyto-protective enzymes [13]. UVA-treated human dermal fibroblasts containing licochalcone A showed higher glutathion levels and a reduced concentration of UV-generated reactive oxygen species [12] (LOE-D). These in vitro data are supported by a double-blind, placebo-controlled in vivo study with 22 healthy volunteers that were treated with either licochalcone A or the vehicle on their volar forearms twice daily for two weeks. The test areas were then irradiated with UVA, and luminescence of the skin was measured with ultra-weak photon emission detection. In the licochalcone A treated skin area, the generation of UVA-induced photons was significantly lower than on the placebo-treated area [12] (LOE-A).

### 6.8. Pine Bark (Pinus pinaster, Ailton)

A standardized polyphenol extract obtained from the bark of *Pinus pinaster (Syn.: P. maritima)* with antioxidant properties has potent photo-protective and pigmentation modulating properties [73]. The effect of pine bark extract on melasma hyperpigmentation was investigated in an open label clinical study on 30 women with melasmatic hyperpigmentations. The subjects received 25 mg pine bark extract three times daily over four weeks. The degree and area of pigmentation was reduced in 80% of the test persons [74] (LOE-B).

### 6.9. Gotu Kola (Centella asiatica (L.) Urban)

*Centella asiatica* contains a variety of pentacyclic triterpenoids, including asiaticoside, brahmoside, asiatic acid, and madecassic acid. Other constituents include centellose, centelloside, and madecassoside. A cream with *Centella asiatica*-extract, tocopherol and a collagen-elastin hydrolysate was tested in a randomized placebo-controlled, double-blind study on 80 pregnant women. The cream was applied daily from the end of the 12^th^ week of pregnancy until delivery. The active cream was significantly superior to the placebo with regard to the number and severity of stretch marks [75] (LOE-A).

Another vehicle-controlled half-side comparison study investigated the effect of a cream containing 0.1% asiaticoside on periocular wrinkles in 27 women. The women applied the creams twice daily over 12 weeks. Silicone imprints revealed a significantly superior reduction of the crow’s feet wrinkles with the asiaticoside cream compared to placebo [76] (LOE-B).

## 7. Wound Healing

Wound healing is a natural physiological response to tissue injury and involves a complex interplay between numerous cell types (keratinocytes, fibroblasts and immune cells), cytokines and the vascular system to stop bleeding, kill bacteria and initiate re-epithelialization. Most herbal remedies traditionally used for wound healing have not been investigated in controlled clinical studies [77]. In contrast, the wound-healing properties of a betulin rich extract from the bark of white birches have been thoroughly investigated. This extract allows the production of a solid phase stabilized emulsion without conventional emulsifiers or preservatives [78] and will be described in more detail.

### 7.1. Birch Bark (Betula spp.)

The wound healing properties of betulin have been elucidated at the molecular level and positively affect all three phases of wound healing (the inflammatory phase as well as migration and differentiation phase of keratinocytes) [79]. The first clinical evidence for the wound healing properties of betulin was achieved in a split thickness wound study with the topical application of a water-free betulin oleogel [80]. Subsequently, several multicentric, controlled, randomized clinical studies on superficial wounds and second degree burns were performed with betulin oleogel [81,82,83] (LOE-A). In 2016, the European Medical Agency (EMA) approved betulin oleogel as a drug for the topical treatment of superficial wounds and burns [84].

### 7.2. Onion (Allium cepa (L.))

A systematic review published in 2017 on anti-scarring agents mentions many positive outcomes in scarring management with onion extract [85].

In a randomized placebo controlled study on 58 subjects that underwent small surgical procedures of the skin, such as excision of skin tumors or punch biopsies, the effect of onion extract on scar formation was investigated. After initial primary wound healing for three weeks, the patients received onion extract or placebo twice daily for 10 weeks. Onion extract significantly improved redness, smoothness, texture and general appearance of the scars compared to placebo [86] (LOE-A).

## 8. Rosacea

Rosacea is an inflammatory skin disease of the face affecting both sebaceous glands and small superficial skin vessels. Different clinical forms of rosacea include an erythematous, papulopustulous and telangiectatic variant. A recent systematic review analyzed the efficacy of herbal medicinal products for the treatment of rosacea [87].

### 8.1. Green Tea (Non-Fermented Camellia sinensis (L.))

Green tea extract contains high amounts of oligomeric proanthocyanidins such as epigallocatechin-3-gallate (EGCG)—a potent antioxidant with photo-protective properties. It has been shown that EGCG inhibits the expression of the vascular endothelial growth factor VEGF and the hypoxia-induced factor 1α (HIF-1α) that both stimulate angiogenesis in the skin. In a very small randomized, double blind, vehicle controlled split face trial four volunteers used a 2.5% EGCG containing cream twice daily over 6 weeks. However, no reduction in skin erythema could be detected, neither clinically nor histologically [88] (LOE-D). However, larger clinical studies are needed to determine if EGCG has a clinically relevant effect. As sun exposure is a trigger factor of rosacea, green tea products might have a relevant therapeutic role via their photo-protective effects.

### 8.2. Licorice Root (Glycyrrhiza inflata, Batalin)

An open label study with 62 volunteers showed that licochalcone A, a flavonoid from licorice root, incorporated in different vehicles significantly reduces erythema in rosacea when applied once daily over 8 weeks. When combined topically with the antibiotic metronidazole it was also well tolerated in 25 patients [89] (LOE-B). The combination of licochalcone A with trans-4-t-butylcyclohexanol, an inhibitor of the cation channel TRPV1, was also investigated. The TRPV1 channel in the skin is known to mediate sensation of pain, itch and warmth. In keratinocytes stimulation of TRPV1 results in increased Ca^2+^-influx, which induces finally cell death and disruption of the epidermal barrier [90]. In an open, not placebo controlled, international multi-center study 1221 patients with sensitive skin prone to redness and rosacea were treated with this combination. An improvement of the studied symptoms (e.g., redness and erythema) could be shown after 4 weeks with applications twice a day [90] (LOE-B). The product was well tolerated. However the used concentration of trans-4-t-butylcyclohexano and licochalcone A was not mentioned in the publication.

### 8.3. Bitter Wood (Simarouba amara Aubl.)

In a recent review on herbal products for rosacea [87] a 4% extract from *Simarouba amara* was the only herbal product that reduced telangiectasia. In a study with 30 patients suffering from rosacea a topical gel with *Simarouba amara*-extract was applied twice daily over 6 weeks. All clinical parameters such as flush, erythema, telangiectasia, papules and pustules were significantly reduced after 6 weeks. No side effects such as pruritus, edema or stinging were observed. The clinical overall improvement was similar to *conventional* standard treatments such as metronidazole or azelaic acid. However, no control group was included in this study [91] (LOE-B).

### 8.4. Tormentil (Potentilla erecta (L.))

Although no clinical trials with tormentil have been conducted in rosacea, recent studies point to a potent vasoconstrictory effect of tormentil that might be useful in treating telangiectasia in rosacea. In a randomized, prospective, placebo controlled double blind patch test with 40 healthy volunteers 1% hydrocortisone and a tormentil extract containing the ellagitannin agrimoniin displayed both a similar blanching effect, that means a paling of the skin [18] (LOE-B). The exact mechanism responsible for the blanching effect is not entirely clear, but local vasoconstriction of smooth muscle cells and consecutive blood flow reduction seem to be involved in this phenomenon [92]. Tormentil-extract displays this vasoconstrictive property at least partly by radical scavenging of NO, an important vasodilator of dermal blood vessels and by inhibition of the endothelial NO-syntase (eNOS) that is constitutively active. Because of its vasoconstrictive effect PE might be beneficial to treat rosacea erythematosa (LOE-C). Facial reddening in rosacea is at least partly mediated by formation of NO via iNOS that is induced by endogenous or exogenous factors such as demodex mites. Sauermann and colleagues showed that the treatment of rosacea with the topical NO inhibitor L-NAME (1% L-NAME cream, applied twice daily for 3 weeks) resulted in a significant reduction of erythema in rosacea patients [93] (LOE-B). However, a clinical study to prove that tormentil extract indeed reduces the facial reddening in rosacea is still missing.

## 9. Acne Vulgaris

Acne vulgaris is characterized by hyperactive sebaceous glands, epidermal hyperproliferation and perifollicular inflammation. The most important pathogens linked to acne-prone skin are for example *Propionibacterium acnes* (*P. acnes*) and *Staphylococcus aureus (S. aureus)*. Botanical and phytochemical options for acne vulgaris therapy are summarized in a systematic review published in 2014 [94].

### 9.1. Tea Tree (Melaleuca alternifolia (Maiden & Betche) Cheel)

In a single-blind, randomized study 5% tee tree oil was compared to 5% benzoyl peroxide on 124 acne patients in topical application. After 3 months of treatment signs and symptoms had markedly improved with both preparations. There were no differences between the two therapies [95] (LOE-B). A vehicle-controlled, randomized, double-blind study with 60 acne patients confirmed the efficacy of a gel containing 5% tea tree oil when applied twice daily over 45 days [96] (LOE-A).

### 9.2. Green Tea (non-fermented Camellia sinensis (L.))

It could be shown that epigallocatechin-3-gallate (EGCG), the major polyphenol in green tea, displays apoptotic, sebosuppressive and anti-inflammatory effects on human sebocytes. Furthermore it displays antibacterial effects on *P. acnes* [97]. EGCG significantly improved acne in an 8-week randomized, split-face, clinical trial with 35 patients that were treated with 1% or 5% EGCG solution twice daily [97] (LOE-A). The efficacy of a lotion with 2% green tea extract was demonstrated in a prospective, non-randomized study on 20 patients with acne using the preparation twice daily for 6 weeks [98] (LOE-B).

### 9.3. Hop (Humulus lupulus (L.))

Hop extract shows anti-oxidative and anti-inflammatory effects. In the microdilution test hop extract inhibited the growth of *P. acnes* and *S. aureus* already at a concentration of 3.1 and 9.4 µg/mL respectively. In addition, a gel formulation with 0.3% hop extract (*w*/*w*) showed antibacterial activity in the agar diffusion test against *P. acnes* and *S. aureus* (inhibition zone value: 5.5 mm and 3 mm, respectively) (LOE-C). Therefore, hop extract might be an alternative treatment option for acne-prone skin [99] and should be tested in clinical studies.

## 10. Conclusions

Botanical compounds such as salicylic acid, methoxsalen and chrysarobin have been traditionally used and still play an important role in the treatment of psoriasis. Recently, the alkaloid indirubin from indigo has been shown to be effective against psoriasis in randomized clinical trials. Glycyrrhetinic acid and licochalcone A from licorice have been shown to be effective in the treatment of atopic dermatitis. The toxic diterpene ester ingenol mebutate from petty spurge has been approved as a highly effective prescription drug for the treatment of superficial epithelial skin cancer (actinic keratoses). Only recently, betulin-oleogel obtained from birch bark has been approved as a drug for the topical treatment of superficial wounds and burns. These examples illustrate that botanical compounds and extracts that are summarized in Table 1 have a great potential to be developed as prescription or over-the-counter drugs in dermatology.

## Figures and Tables

**Table 1:** Summary of herbal compounds for the treatment of skin diseases.

Disease	Plant	Compound	Treatment	L O E	Ref
Atopic dermatitis	St. John’s wort *(Hypericum perforatum* (L.)*)*	Hyperforin 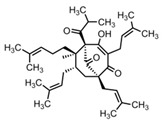	- Ointment with 1.5% hyperforin twice daily for 4 weeks	A	[6]
	Licorice (*Glycyrrhiza glabra* (L.))	Glycyrrhetinic acid 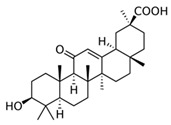	- Hydrophilic ointment with 2% glycyrrhetinic acid three times daily for 5 weeks	A	[15]
			- Cream with 0.6% glycyrrhizinic acid and licorice extract (0.1% *Glycyrrhiza uralensis* root extract) twice daily for 2 weeks	B	[17]
		Licochalcone A 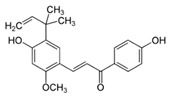	- Cream with < 1% licochalcone A	A	[16]
	Tormentil (*Potentilla erecta* (L.))	Tannins	- Cream with 2% tannins from the rhizome of tormentil for 48 h in an UV-erythema test	B	[18]
			- Cream with 2% tannins from the rhizome of tormentil twice daily for 2 weeks	B	[19]
	Bitter substances	Gentian extract	- Cream with 5% gentain extract twice daily for 4 weeks increases in volunteers epidermal lipids that are reduced in atopic dermatitis	B	[21]
Atopic dermatitis	Evening primrose *(Oenothera biennis* (L.)*)*	Primrose oil	- Meta-analysis for the use of primrose oil	A	[22]
Psoriasis vulgaris	Araroba tree *(Vataireopsis araroba* (Aguiar) Ducke)	Dithranol 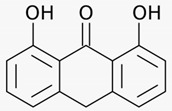	- Daily short treatments (15 - 45 min) with dithranol cream of stepwise rising concentrations up to 5% for 12 weeks	A	[26]
	Lace flower (*Ammi majus* (L.) and *Ammi visnaga* (L.))	8-methoxypsoralen (8-MOP) 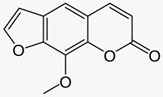	- Oral 8-MOP PUVA (0.6 mg/kg) twice per week	A	[27]
			- Bath PUVA with 5 mg/l 8-MOP 4 times per week	A	[28]
			- Cream PUVA with 0.1% 8-MOP 12 treatments	A	[29]
		5-methoxypsoralen (5-MOP) 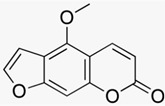	- Oral 5-MOP PUVA (1.2 mg/kg) twice per week	A	[27]
	Barberry bark (*Mahonia aquifolium* (Pursh) Nutt.)	Mahonia ointment	- 10% Mahonia ointment twice daily for 12 weeks	A	[30]
	Indigo (*Baphicacanthus cusia* Brem.)	Indigo naturalis containing indigo and indirubin 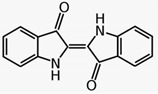	- 10% indigo ointment (1.4% indigo and 0.16% indirubin) daily for 12 weeks	A	[31]
Psoriasis vulgaris		Indigo extract	- Indigo ointment (50 μg/g and 200 μg/g indigo extract) applied twice daily over 8 weeks	A	[33]
	Turmeric (*Curcuma longa* (L.))	Curcumin 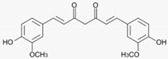	- No randomized placebo-controlled studies available, only in vitro studies or in vivo studies using mice		
	Olibanum (*Boswellia serrata,* Triana & Planch)	3-O-Acetyl-11-keto- β-boswellic acid 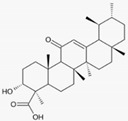	- Olibanum ointment containing 5% 3-O-Acetyl-11-keto-β-boswellic acid three times daily for 12 weeks	B	[39]
	St. John’s wort (*Hypericum perforatum* (L.))	St. John’s wort extract	- 5% St. John’s wort extract twice daily for 4 weeks	B	[41]
			- 5% St. John’s wort extract once daily for 4 weeks	B	[42]
Herpes simplex	Lemon balm (*Melissa officinalis* (L.))	Lemon balm extract	- Cream containing 1% dried *Melissa officialis* extract applied 4 times daily for 5 days	A	[46]
	Sage (*Salvia officinalis* (L.)) and Rhubarb (*Rheum palmatum* (L.))	Sage and rhubarb extract	- Treatment with cream containing sage extract (23 mg/g) or combination of sage and rhubarb extract (23 mg/g each) until healing (7.6 days with sage and 6.7 with combination)	A	[47]
Actinic keratoses	Birch bark (*Betula spp*.)	Betulin 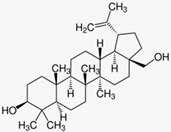	- Treatment with betulin oleogel for 3 months without effect in high grade actinic keratoses	A	[49]
			- Birch bark ointment (betulin oleogel) for 2 months	B	[50]
			- Betulin oleogel for 3 months	B	[51]
	Petty spurge (*Euphorbia peplus* (L.))	Ingenol mebutate 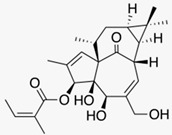	- Gel with 0.025% or 0.05% ingenol mebutate daily for 3 days	A	[53]
			- Gel with 0.0025%, 0.01% or 0.05% ingenol mebutate on two consecutive days or on day 1 and day 8	A	[54]
Photo-protection and esthetic dermatology	Green tea (*Camellia sinensis* (L.))	Epigallocatechin-3-gallate 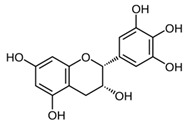	- 1 mg/cm^2^ skin of epigallocatechin-3-gallate before UV exposure	D	[56]
	Dyer’s weed (*Reseda luteola* (L.))	Luteolin 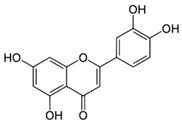	- Dyer’s weed extract containing 2.5% luteolin	A	[61]
		Dyer’s weed extract	- Cream with 0.1% Dyer’s weed extract, 0.1% tocopherol and 0.05% ubiquinone three times daily for 7 days	A	[63]
	Cocoa tree (*Theobroma cacao* (L.))	Flavanol 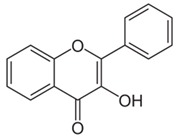	- Ingestion of cocoa with high (326 mg/d) or low (27 mg/d) flavanol content in water over 12 weeks	A	[64]
Photo-protection and esthetic dermatology	Carotinoids	β-carotene 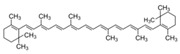	- 24 mg β-carotene orally daily for up to 8 weeks	A	[65]
		Lycopene 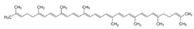	- 10 mg lycopene daily for 12 weeks	B	[66]
			- Tomato paste containing 16 mg lycopene daily for 12 weeks	B	[67]
	Citrus fruits (*Citrus* spp.)	Limonene 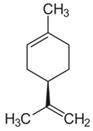	- Regular citrus fruit consumption with or without peel	C	[68]
	Coffee plants (*Coffea* spec.)	Caffeine 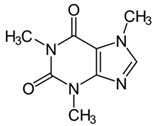	- Observatory studies on caffeine intake	B B	[69] [70]
	Licorice (*Glycyrrhiza glabra* (L.))	β-glycyrrhetinic acid 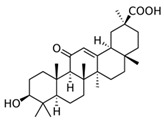	- Ointment with 2.5% β-glycyrrhetinic acid twice daily over 4 weeks	A	[71]
		Glabridin 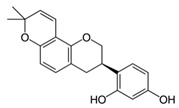	- Ointment with 0.5% glabridin on guinea pig skin	D	[72]
Photo-protection and esthetic dermatology		Licochalcone A 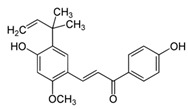	- 9 μM Licochalcone A in in vitro experiments	D	[12]
			- Ointment containing licochalcone A twice daily for 2 weeks	A	[12]
	Pine bark (*Pinus pinaster* Ailton)	Pine bark extract	- Oral ingestion of 25 mg pine bark extract three times daily for 30 days	B	[74]
	Gotu kola (*Centella asiatica* (L.) Urban)	Gotu kola extract	- Cream with Centella asiatica extract applied daily from the end of the 12th week of pregnancy until delivery	A	[75]
		Asiaticoside 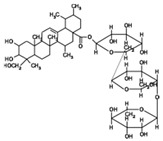	- Cream containing 0.1% asiaticoside twice daily for 12 weeks	B	[76]
Wound healing	Birch bark (*Betula* spp.)	Betulin 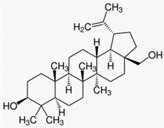	- Wounds including second degree burns and split-thickness skin graft wounds were treated with betulin oleogel (containing 10% betulin) till wound closure or up to 28 days	A	[81]
	Onion (*Allium cepa* (L.))	Onion extract	- Onion extract twice daily for 10 weeks after initial primary wound healing	A	[86]
Rosacea	Green tea(*Camellia sinensis* (L.))	Epigallocatechin-3-gallate 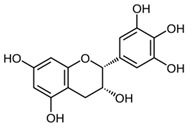	- Cream containing 2.5% epigallocatechin gallate twice daily for 6 weeks	D	[88]
	Licorice root (*Glycyrrhiza inflata* Batalin)	Licochalcone A 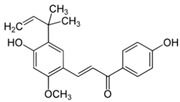	- Licochalcone A in different vehicles applied daily for 8 weeks	B	[89]
			- Treatment with licochalcone A and trans-4-t-butylcyclohexanol (TRPV1 inhibitor) twice a day for 4 weeks	B	[90]
	Bitter-wood (*Simarouba amara* Aubl.)	Bitter-wood extract	- Gel containing 4% *Simarouba amara* extract twice daily for 6 weeks	B	[91]
	Tormentil (*Potentilla erecta* (L.))	Tormentil extract	- Tormentil extract (5%) applied topically once for 48 h to volunteers in a patch test	C	[18]
Acne vulgaris	Tea tree *(Melaleuca alternifolia* (Maiden & Betche) Cheel)	Tea tree oil	- Gel containing 5% *Melaleuca alternifolia* oil applied topically for 3 months	B	[95]
			- Gel containing 5% *Melaleuca alternifolia* oil applied twice daily for 45 days	A	[96]
	Green tea *(Camellia sinensis* (L.)*)*	Epigallocatechin-3-gallate 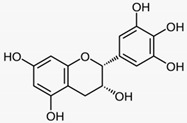	- Treatment with 1% or 5% epigallocatechin-3-gallate solution twice daily for 8 weeks	A	[97]
Acne vulgaris		Green tea extract	- Lotion with 2% *Camellia sinensis* extract twice daily for 6 weeks	B	[98]
	Hop *(Humulus lupulus* (L.)*)*	Hop extract	- 3.1 μg/mL and 9.4 μg/mL of *Humulus lupulus* extract in vitro	C	[99]
			- Gel containing 0.3% *Humulus lupulus* extract in vitro		
